# Long‐term changes in bat activity in Quebec suggest climatic responses and summer niche partitioning associated with white‐nose syndrome

**DOI:** 10.1002/ece3.6194

**Published:** 2020-06-02

**Authors:** Julie Faure‐Lacroix, André Desrochers, Louis Imbeau, Anouk Simard

**Affiliations:** ^1^ Centre d'étude de la forêt Faculté de foresterie de géographie et de géomatique Université Laval Québec City QC Canada; ^2^ Centre d'étude de la forêt UQAT Rouyn‐Noranda QC Canada; ^3^ Ministère des Forêts de la Faune et des Parc du Québec Quebec Centre for Biodiversity Science Québec City QC Canada

**Keywords:** Chiroptera, climate, monitoring transects, white‐nose syndrome

## Abstract

In North America, the greatest and most sudden threat to hibernating bats is white‐nose syndrome (WNS), which has caused massive declines in populations since 2006. Other determinants of bat dynamics, such as the climate, and the effect of reduction in the number of individuals sharing foraging space and summer roosting habitat may have an effect on population dynamics. We analyzed transect acoustic bat surveys conducted with ultrasonic detectors in 16 regions in Quebec, Canada, between 2000 and 2015. We used piecewise regression to describe changes in activity over time for each species and a meta‐analytic approach to measure its association with the North Atlantic Oscillation (NAO). As expected, mouse‐eared bat (*Myotis* spp.) activity sharply declined after the onset of WNS, down by 79% after 3 years. In contrast, big brown/silver‐haired bat activity increased over the same period, possibly due to a release of competition. Hoary bats and red bats remained present, although their activity did not increase. *Myotis* activity was positively correlated with a one‐year lag to the NAO index, associated with cold conditions in winter, but warm autumns. Big brown/silver‐haired and hoary bats were also more active during NAO‐positive years but without a lag. We conclude that combinations of threats may create rapid shifts in community compositions and that a more balanced research agenda that integrates a wider range of threats would help better understand and manage those changes.

## Graphical abstract

We analyzed 10 years of acoustic bat monitoring data from 16 regions in the province of Quebec, Canada, before and after the onset of white‐nose syndrome (WNS). We found a general response to climate for some species, a sharp decline in activity for the species affected by WNS, and subsequently an apparent relaxation of niche partitioning between large, high‐flying species and small, low‐flying species.

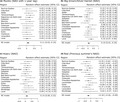

## 1 INTRODUCTION

In North America, one of the most sudden collapses of mammal populations on record is that of hibernating bat populations, caused by white‐nose syndrome (WNS, Frick, Pollock, et al., 2010). After the initial discovery of a bat population contaminated by *Pseudogymnoascus destructans* (*Pd*; *Ascomycota*) in the State of New York (USA), over 5.5 million bats had died from WNS annually (Froschauer & Coleman, 2012), with a regional mean of mortality rates of 73% (Frick, Pollock, et al., 2010). *Pd* is a non‐native psychrophilic fungus that preferentially develops at temperatures between 4°C and 20°C, which are commonly measured in bat hibernacula during winter (Blehert et al., 2009; Gargas, Trest, Christensen, Volk, & Blehert, 2009; Minnis & Lindner, 2013). The species most affected are the mouse‐eared bats (*Myotis* spp.), including the little brown bat (*M. lucifugus*) and Northern long‐eared bat (*M. septentrionalis*), as well as tricolored bats (*Perimyotis subflavus*)*.* Big brown bats (*Eptesicus fuscus*) share hibernacula with *Myotis* species and have also been diagnosed with WNS (Blehert et al., 2009). The latter is sometimes depicted as resistant to *Pd* infection (Bernard, Foster, Willcox, Parise, & McCracken, 2015), but new evidence suggests tolerance rather than resistance to *Pd* (Dorville, 2019); in any case, their populations have not suffered the same loss as other hibernating species.

Migratory bats such as the eastern red bat (*Lasiurus borealis*), the silvered‐haired bat (*Lasionycteris noctivagans),* and the hoary bat (*Lasiurus cinereus*) do not hibernate in caves, and thus, they are not directly threatened by WNS (Bernard et al., 2015). However, they are vulnerable to other threats, most notably collisions with wind turbines (Arnett et al., 2008). The effect of wind turbines is considered to be large enough that it could cause hoary bat populations to decline by as much as 90% in the next 50 years, from an estimated initial population of 2.5 million (Frick et al., 2017). Bats are also vulnerable to pesticides, as well as to habitat loss and fragmentation, which are known to have caused steady population declines in migratory bats over the past few decades, most notably in red bats (Carter et al., 2003; Whitaker, Brack, & Cope, 2002; Winhold, Kurta, & Foster, 2008).

When some species decline, others that share the same resources might benefit from a competitive release (Larsen, 1986; Ruscoe et al., 2011). Insectivorous bats are known to compete for foraging resources (Carter, Menzel, Chapman, & Miller, 2004; Hickey, Acharya, & Pennington, 1996), as well as hibernating, reproductive, and roosting sites (Perkins, 1996; Thalken, Lacki, & Johnson, 2018). Thus, we expect that a competitive release has occurred in insectivorous bats following the onset of WNS (Jachowski et al., 2014), which would allow species like big brown bats to exploit the portion of their diet that is shared with little brown bats, as found by Morningstar, Robinson, Shokralla, and Hajibabaei (2019) in Ontario. Additionally, O’Keefe, Pettit, Loeb, and Stiver (2019) found that the patterns in hibernacula mirrored the decline in *Myotis*, as well as the stability in big brown bat summer populations. It could mean that big brown bats would benefit from a competitive release for roosting locations in the long term, as well as increased availability of foraging habitat, resulting in better survival and reproduction.

Besides catastrophic events like the WNS, bats probably respond to subtle impacts of weather and climate, as exemplified by the association between Leisler's bat *(Nyctalus leisleri*) activity and weather in spring and winter (Schorcht, Bontadina, & Schaub, 2009). For all species, a warm autumn with more insects could have a positive impact on reproduction, hibernation, and first‐year survival, as it is the season during which mating and prehibernation fat accumulation occurs (Ewing, Studier, & O’Farrell, 1970; Kunz, Wrazen, & Burnett, 1998) and particularly for juveniles (Kunz et al., 1998). For hibernating species, mild winters with little temperature fluctuations would reduce energy expenditures for bats which choose to roost near cave entrances rather than in deeper, more stable locations (Brack, 2007).

In the Province of Quebec (Canada), bats have been monitored annually since 2000 using recorded detections on motorized acoustic transects, that is, prior to and following the onset of WNS (Jutras, Delorme, Mc Duff, & Vasseur, 2012). We provide a first in‐depth analysis of those surveys, examining to what extent WNS is temporally associated with bat populations, and how yearly changes in activity are driven by weather as modulated by climatic variables. We examine six predictions: (a) that *Myotis* species decline from the onset of WNS, consistent with estimates from the USA (Frick, Pollock, et al., 2010); (b) that such declines create a release from competition for foraging space thus leading to a relative increase in the activity of species unaffected by WNS; (c) that migratory bats decline with the creation of new wind energy facilities; (d) that stochastic fluctuations in bat activity are modulated by climatic variables; (e) that years with long and warm falls would benefit all bat species and result in an increased recorded activity the following summer; and (f) that mild winters are associated with high hibernating bat activity in the following summer for species at risk of hibernating in unstable conditions in caves.

## 2 MATERIALS AND METHODS

### 2.1 CHIROPS network

**FIGURE 1:**
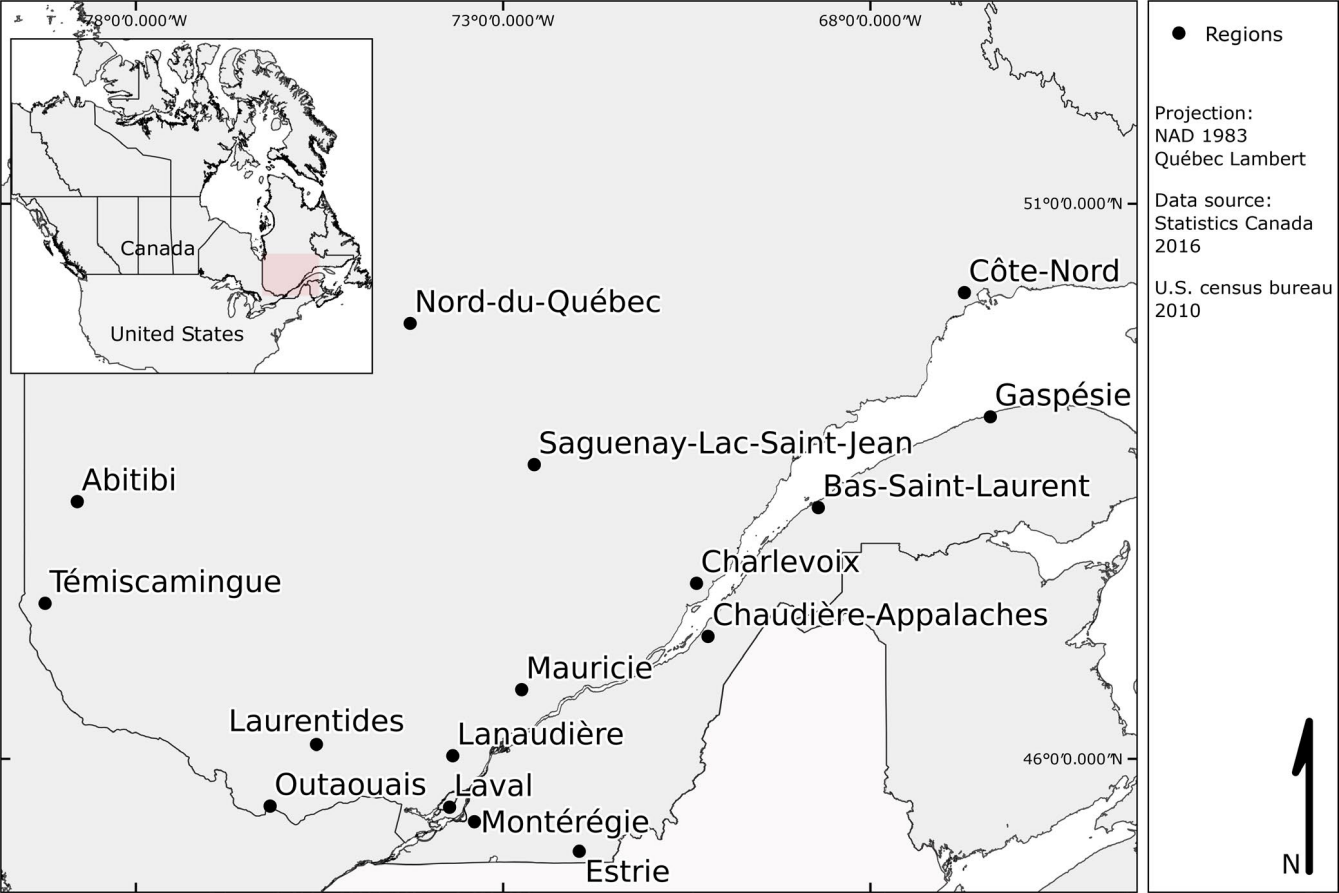
Location of motorized recording transects monitoring bat activity as of 2015 in Quebec, Canada. A description of each administrative region can be found in Table 1

The Quebec network for acoustic bat monitoring, known as CHIROPS (Jutras et al., 2012), relies upon volunteers, technicians, and biologists from the provincial wildlife ministry (Ministère de la Faune, des Forêts et des Parcs; MFFP). At its inception in 2000, the network consisted of three listening transects using existing roads and included nine transects by 2009. In 2015, 16 regions in the province of Quebec participated in the survey (Figure 1, Table 1). The group located the transects in a forested area when possible (one exception being Laval, which is located in the city) representative of the type of forest typical of the area (see Table 1) and included preferred habitats for, such as wetlands or watersheds. They usually selected them following a year of assessment of multiple potential transects for the same area, after which they chose the candidate deemed most suitable for bat recording based on the preselection results. Selected transects had to be about 20 km in length, in an approximate triangular, U‐ or 8‐shaped form, and located on quiet and unfrequented roads. Each spring, the participants were assigned a detection kit consisting of an ultrasonic detection device (see *Transect Monitoring*) and a GPS receiver.

**Table 1:** Description of each participating region of the CHIROPS acoustic bat surveys, Quebec, 2000–2015

Region	Lat/long	First year of monitoring	First assessment of WNS	Common habitat	Distance from nearest weather station (km ± *SE*)	Distance from closest route (km)	Distance from other routes (km ± *SE*)	Mean number of visits (±*SE*)
Bas‐Saint‐Laurent	N48°19′/W68°42′	2002	2014	Agricultural, forest	27.7 ± 0.6	145	432.1 ± 51.8	2.2 ± 0.7
Saguenay‐Lac‐Saint‐Jean	N48°42′/W72°34′	2002	2012	Agricultural, forest	33.5 ± 0.5	185	353.3 ± 27.1	4.4 ± 0.2
Capitale‐Nationale	N47°38′/W70°22′	2002	2012	Agricultural, forest	41.0 ± 0.3	56	351.5 ± 42.9	2.8 ± 0.3
Mauricie	N46°39′/W72°44′	2000	2011	Forest, agricultural	10.5 ± 0.3	100	307.6 ± 41.9	3.1 ± 0.5
Estrie	N45°07′/W71°57′	2000	2010	Agricultural	30.4 ± 0.2	116	384.9 ± 51.1	3.4 ± 0.3
Outaouais	N45°33′/W76°10′	2003	2010	Urban, agricultural, mixed forest, deciduous forest	48.2 ± 0.4	80	420.9 ± 59.8	2.8 ± 0.4
Témiscamingue	N47°27′/W79°14′	2003	2011	Agricultural, deciduous forest, coniferous forest, mixed forest	78.0 ± 0.3	108	556.2 ± 61.0	3.9 ± 0.2
Abitibi	N48°22′/W78°47′	2003	2011	Agricultural, deciduous forest, coniferous forest, mixed forest, wetlands	22.9 ± 0.5	108	542.9 ± 55.3	3.8 ± 0.2
Côte‐Nord	N50°13′/W66°43′	2002	never	Coniferous forest	32.9 ± 0.4	125	613.9 ± 63.0	3.1 ± 0.4
Gaspésie	N49°08′/W66°22′	2002	2015	Mixed forest, deciduous forest, coniferous forest, agricultural	21.5 ± 0.3	125	582.0 ± 64.4	2.8 ± 0.3
Nord‐du‐Québec	N49°57′/W74°15′	2003	2012	Mixed forest, coniferous forest, deciduous forest	52.8 ± 0.6	185	449.5 ± 25.1	3.5 ± 0.3
Chaudière‐Appalaches	N47°08′/W70°12′	2002	2012	Mixed forest, agricultural	28.4 ± 0.3	56	356.7 ± 44.0	3.9 ± 0.3
Lanaudière	N46°01′/W73°41′	2006	2011	Urban, agricultural, mixed forest, coniferous forest	28.0 ± 0.3	54	320.9 ± 52.2	2.8 ± 0.5
Laurentides	N46°08′/W75°32′	2004	2011	Mixed forest, coniferous forest, agricultural	38.2 ± 0.3	80	370.1 ± 55.1	2.6 ± 0.2
Montérégie	N45°24′/W73°23′	2012	2010	Urban, agricultural, deciduous forest	9.4 ± 0.2	30	348.5 ± 56.5	3.5 ± 0.5
Laval	N45°32′/W73°43′	2000	2010	Urban	9.1 ± 0.2	30	342.0 ± 56.8	4.2 ± 0.4

Note

First assessments of WNS years are based on reports of affected individuals that are provided by the provincial Ministère de la Faune, des Forêts et des Parcs (MFFP), or estimates based on nearby regions. All census data are from 2011 and are provided by (Statistics Canada, 2012).

### 2.2 Description of the regions

In the southwestern part of the province, transects were mostly located outside of a city, except for the one located in Laval. This area is densely populated, and agricultural lands are often present along the transects that were surveyed. The Montérégie transect was added in 2012 to compensate for the habitat loss that occurred in the Laval transect over the years. In the northwest (47° N, 74° W), the landscape is more heavily forested, particularly in the Nord‐du‐Québec administrative region. Northeastern transects were located near small‐ to medium‐sized towns with a large proportion of fields and some forest stands. In the southeast, transects were located mostly in rural areas.

**FIGURE 2:**
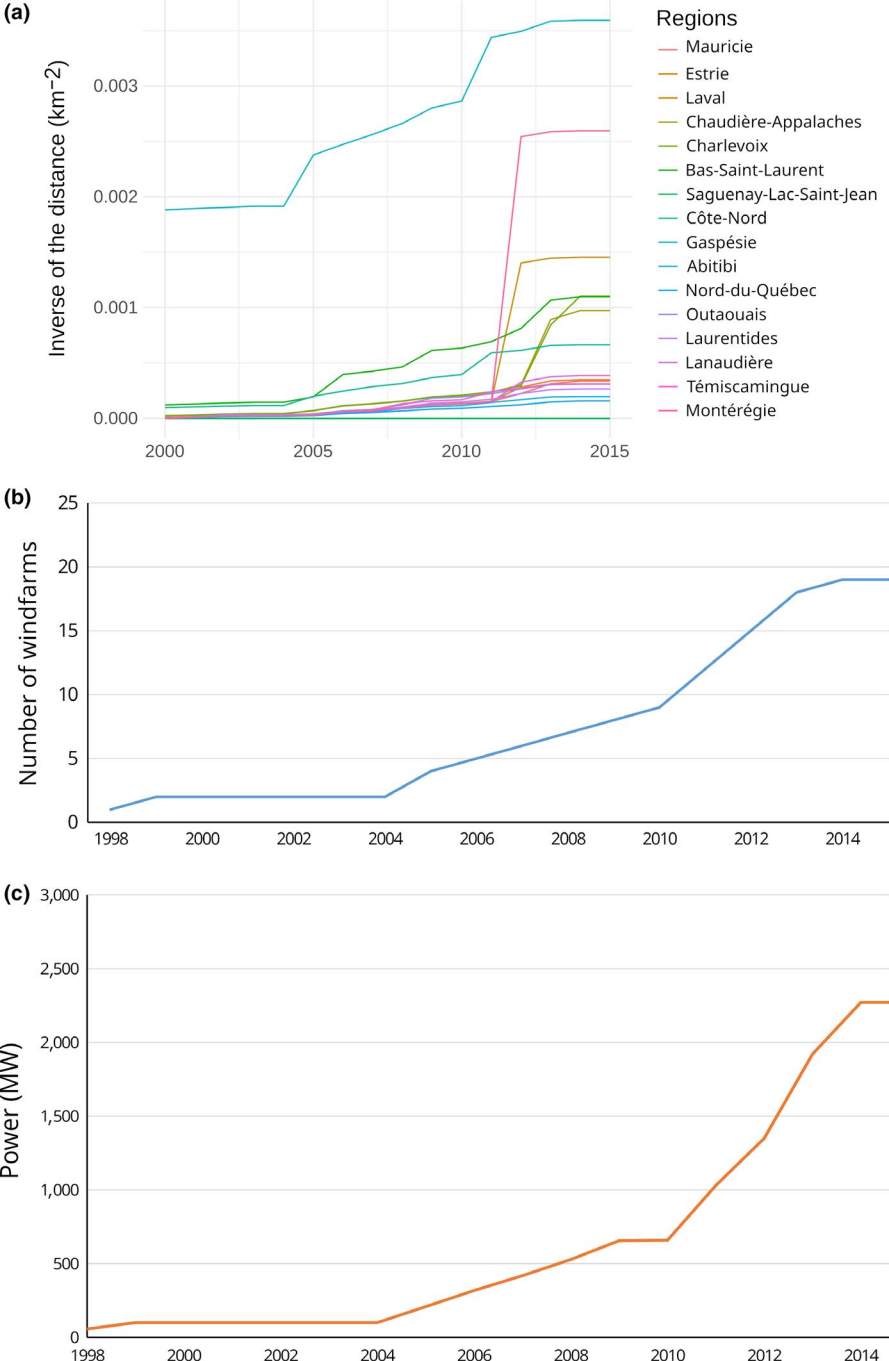
Proximity of each region to wind energy facilities. a) The curves are the sum of the inverse of the squared distance of all facilities to each transect centroid, b) cumulative number of windfarms in the province, and c) cumulative theoretical power (megawatts) of the windfarms

Wind energy facilities existed in the province before 2000, but the industry has grown more rapidly, especially since 2011 (Figure 2). The proximity to a wind energy facility increased for most transects, but it mostly affected northeastern transects that were located in the regions of Gaspésie, Charlevoix, Chaudière‐Appalaches, Côte‐Nord, and Bas‐Saint‐Laurent.

### 2.3 Transect monitoring

During monitoring nights, participants recorded echolocation calls using an ultrasonic detector (AnaBat; Titley Scientific) coupled to a tape recorder. From 2012, Anabats were replaced by Anabat II, which allowed automatic recording on a CF card rather than manual recording on a cassette. Participants conducted CHIROPS surveys between 15 June and 31 July of each year (mean: 194th day of the year, *SE* = 0.15). Most years, the mean sampling date fell between 10 July and 14 July, except for 2001 (mean = 9 July), 2002 (4 July), and 2008 (16 July). We kept the surveys that were completed and excluded any survey that was conducted outside of the allowed date period. They were instructed to select a maximum of 4 nights with no precipitation, wind speeds below 5 km/h, and a temperature that was at or above the local seasonal mean. The mean sampling effort per transect is detailed in Table 1. In each region, a survey team traveled in a vehicle at 20 km/h. After completing the 20‐km‐long transect once, they drove back to the beginning of the transect to sample it a second time, for a total of 40 km. When a call was detected, participants remained stationary for 2 minutes while pointing the detector toward the bat. This maneuver originally helped to improve call quality from the Anabats, which were coupled to a cassette recorder and, therefore, identification accuracy. The transects typically began 15 min after sunset (mean starting time 9:20 p.m., *SD* = 28 min) and ended 2 hr 35 min later in average (*SD* = 42 min), which is well within the period of peak activity found in the province of Quebec for all species (Faure‐Lacroix, Desrochers, Imbeau, & Simard, 2019).

### 2.4 Climatic data

To assess the influence of climate on bat activity in the province, we used the North Atlantic Oscillation (NAO) index from 1999 to 2015 (National Center for Atmospheric Research Staff, 2018), a periodic index calculated from the difference of the sea‐level surface pressure between the Subtropical High (an area between 35°N and 40°N in the North Atlantic) and the Subpolar Low (over Greenland, Climate Prediction Center, 2012). Although several climate indices can be used according to their geographical influence (see a review by Stenseth et al., 2003), the use of NAO index is generally prevalent in eastern North America given that it has measurable effects on terrestrial species, including amphibians, mammals, and birds (Forschhammer, Post, & Stenseth, 1998). The advantage of NAO is that such a global index is less sensitive to spatial scales than local indices and may relate more strongly to biological effects (Hurrell, 1995). In Canada, positive values of NAO are normally associated with cooler ambient temperatures, northern winds, and dry conditions, whereas negative values are associated with warm and wet conditions (Hurrell, Kushnir, Ottersen, & Visbeck, 2003). To confirm the influence of climatic variations on the local weather, we used data that were recorded at the closest weather stations (Environment Canada, 2016) from 1999 to 2015 (see Table 1).

We calculated several versions of the NAO index: a mean of the 12 months preceding each summer (yearly NAO) and one for each season (autumn, winter, spring, summer). By doing this, we accounted for the specific movements of each group of bats. Even though their specific seasonal movements are largely unknown in the province, it is safe to say that hibernating species remain in Northeastern America over winter even when considering potential seasonal movements between hibernacula and summer roosts ranging from tens to hundreds of kilometers (Neubaum, O’Shea, & Wilson, 2006; Norquay, Martinez‐Nuñez, Dubois, Monson, & Willis, 2013), while migrating species leave during autumn and come back the following year (Cryan, 2003). Consequently, the latter group may only be significantly affected by northeastern conditions as modulated by NAO from the current summer or the previous summer. However, we included the possibility of a one‐year lag to the response to climate conditions. In mid‐June/early July, Myotis give birth to pups which fledge after ca. 6 weeks (Powers, Kandarian, & Kunz, 1991; Wimsatt, 2012), while big brown bats give birth in June and pups take flight 3 to 5 weeks later (Kurta, Kunz, & Nagy, 1990). Big brown pups are likely detected during the late‐June/late‐July surveys that same year, while more of the activity from Myotis pups would be recorded the following year since they start fledging later than big brown bats. A lagged effect of weather on body condition has also been detected in other species, notably through their effect on reproduction (Simard, Huot, de Bellefeuille, & Côté, 2014).

### 2.5 Detections and bat activity

We assessed bat activity by counting the number of detected passes for each species using motorized transects. The consulting firm WPS (formerly Envirotel 3,000) manually identified calls and considered a pass to be a minimum of 3 calls per 15‐s file. They identified the calls based on typical shapes of the calls of bats found in the area and known to the consulting firm and rejected those they could not identify because of poor recording quality and we validated the identifications using a DFA analysis (Adams, 2013). Calls of big brown and silver‐haired bats are cryptic, and thus, they were combined in further analyses to minimize errors in identification (hereafter, big brown/silver‐haired). *Myotis* species, which include little brown, northern long‐eared, and eastern small‐footed bats, were also considered as a group to minimize errors. In our area, little brown bats typically account for most detections, followed by northern long‐eared bats; eastern small‐footed bats are rarely detected (MFFP, unpublished). The calls of eastern red bats, hoary bats, and tricolored bats are distinctive enough to be considered separately.

### 2.6 Measuring temporal dynamics within regions

To assess sudden or gradual changes in activity for each species, we conducted piecewise regressions using the *segmented* package (Muggeo, 2015) in R (R Core Team, 2016). Because the transects were resampled twice in the same night and visited multiple times a year, we summed the number of calls for each transect every year, as was done by Crewe, Taylor, and Lepage (2016). Because we used count data and that the sampling effort was of constant distance but various time lengths, we used a generalized linear model function (glm) with a Poisson family and added the log of the sampling effort in minutes as an offset. In piecewise regressions, the independent variable is segmented into a predetermined number of intervals and a separate regression is fitted for each interval, which allows the estimation of “breakpoints,” that is, regressor values at which the relationship changes. For hibernating species, the breakpoint was interpreted as corresponding to the period of contamination by WNS and two intervals that represented the periods pre‐WNS and ongoing WNS. We had access to a list of years of first assessment of WNS in each region, which corresponded to the year when a sick bat was first reported in that area. In some case when no sick bat has been found in a region, the first assessment date has been inferred from the neighboring regions. According to the year of the first assessment of WNS for each region (Table 1), we split regions into two groups: an “early‐onset” group for which the first detection of WNS by regional authorities occurred before 2012; and a “late‐onset” group for which the first detection occurred after 2012 and the one region for which the first signs of WNS were documented in 2015. We assumed two intervals: one covering the years before the onset of WNS and one following the onset of WNS. In the piecewise regressions, we used 2011 as an arbitrary starting point to look for breakpoints (psi value) if the region belonged to the early‐onset group and 2014 if it belonged to the late‐onset group. For migrating species, which should not be affected negatively by WNS, the value of 2011 corresponds to the year before the beginning of the construction of several wind energy facilities (Figure 2). We split the transects into two groups: the northeastern transects (*n* = 5, see methods for the list) closest to the major facilities and the rest of the province (*n* = 11).

### 2.7 Estimating climatic effects

We tested variation in the NAO index on bat activity for each species using meta‐analysis, with each region treated as a separate “study.” A meta‐analysis aggregates data from multiple studies or in this case, multiple sites from a single study. In all sites, each bat species was considered a separate population that could respond differently to several variables, and as it is common in meta‐analyses, we did not work with one single a priori explanatory variable. We detrended variables using linear or LOESS regressions when required (Fox & Weisberg, 2018). We computed Pearson product‐moment correlations (*r*) for each species (Borenstein, Hedges, Higgins, & Rothstein, 2009). Using the *metaphor* package in R (Viechtbauer, 2015), we standardized the Pearson coefficients as *z*‐scores using Fisher's *Z* transform. We used *r*_z_ to calculate the effect size (*E* ^++^), weighted with the reciprocal of the sampling variance for each region. It considered a number of degrees of freedom of 16 or less according to the number of regions included in the analysis. We considered effect sizes as significant if their confidence interval did not include zero. To assess whether the strength of the relationship was modulated by the location of the region, we computed separate group effect sizes (*E* ^+^) for each quadrant in the province (North, East, South, and West), which took into account the number of sites in each group. The number of degrees of freedom was equivalent to the number of regions included in the analysis. We calculated 95% confidence intervals (CI) by bootstrapping with 999 resamples of the data for each group. We computed Cochran's Q heterogeneity test (Cochran, 1954) to evaluate differences in effect sizes within groups (*Q*_E_) and between groups (*Q*_M_). We separated the climatic analysis and the temporal analysis related to WNS and wind energy facilities because we chose to analyze the climatic data as a meta‐analysis, for which the use of beta coefficients is often considered dubious, even though it may be robust enough with a much larger sample size than it is the case in our study (Peterson & Brown, 2005).

## 3 RESULTS

### 3.1 Relationship between white‐nose syndrome and bat activity

*Myotis* was the most frequently encountered group of species prior to the first detection of WNS, but we recorded more big brown/silver‐haired bats activity in the years following the estimated onset of WNS. Before WNS, we recorded a mean (±*SE*) of 34.18 ± 3.11 passes/year for *Myotis*; during the years after the onset of WNS, this count dropped by 79%, that is, down to 7.1 ± 1.55 passes/year. Mean nightly detection for big brown/silver‐haired bats more than doubled from 20.54 ± 2.81 passes/year before WNS to 43.22 ± 5.61 passes/year in the years following the breakpoint. Comparing raw means of activity before and following the breakpoint, red bat activity had remained biologically unchanged from 1.51 ± 0.23 passes/year to 2.35 ± 0.68 passes/year. Hoary bats also remained biologically stable when comparing global mean activity before and after the breakpoint. However, until the beginning of the progress of wind energy facilities in 2012, the mean yearly detection was 30.05 ± 3.23 passes/transect while after that period, the mean number of detections decreased to 23.62 ± 4.61 passes/transect. The mean yearly pass count of tricolored bats before the breakpoint changed from 0.07 ± 0.03 to 2.1 ± 0.3 passes/night following breakpoint. This generally low activity did not allow us to draw strong conclusions regarding the effects of WNS on this group.

Piecewise regression showed that, globally, the levels of bat activity stopped increasing in 2009 (*SE* = 0.74, *p* = .03, Table 2). *Myotis* activity increased throughout the province as a function of the year up until 2010 (*SE* = 0.13, *p* < .001). However, in 2009 (*SE* = 0.13, *p* < .001), the regions where WNS was detected the earliest started experiencing a drop in *Myotis* activity (β = −0.40 ± 0.04, *p* < .001). The effect of WNS measured in early‐onset regions was sufficiently large to reflect the overall activity count for that group, even though the late‐onset regions had yet to show any significant decline of activity. In general, the inclusion or exclusion of the Laval site did not have a major effect on the result, mostly because of the low number of detections.

**Table 2:** Summary of the breakpoints and slopes found in piecewise regression for each species in the province of Quebec, Canada

Group	Species	Breakpoint		Slope before breakpoint [CI]	Slope after breakpoint [CI]
		Year	*SE*		
Early‐onset	Big brown/silver‐haired	2010***	0.29	0.017 [0.003, 0.030]	0.227 [0.200, 0.254]
	Hoary	2012***	0.26	0.066 [0.055, 0.077]	−0.125 [−0.169, −0.081]
	Red	2012***	0.27	0.284 [0.236, 0.333]	−0.686 [−1.014, −0.358]
	Myotis	2009***	0.13	0.074 [0.060, 0.088]	−0.407 [−0.449, −0.364]
	Tricolored	2013***	0.41	0.894 [0.578, 1.210]	−0.006 [−0.227, 0.215]
	Total	2002**	0.86	−0.087 [−0.241, 0.067]	0.038 [0.034, 0.042]
Late‐onset	Big brown/silver‐haired	2005***	0.41	1.042 [0.452, 1.633]	−0.231 [−0.312, −0.15]
	Hoary	*N*/A	—	—	—
	Red	*N*/A	—	—	—
	Myotis	*N*/A	—	—	—
	Tricolored	*N*/A	—	—	—
	Total	2012***	0.49	0.002 [−0.017, 0.021]	−0.430 [−0.672, −0.188]
Overall	Big brown/silver‐haired	2003***	0.18	−0.294 [−0.359, −0.229]	0.133 [0.124, 0.142]
	Hoary	2012***	0.29	0.056 [0.046, 0.066]	−0.116 [−0.16, −0.072]
	Red	2012***	0.30	0.315 [0.268, 0.362]	−0.666 [−0.988, −0.343]
	Myotis	2010***	0.13	0.053 [0.040, 0.066]	−0.364 [−0.400, −0.328]
	Tricolored	2013***	0.42	0.852 [0.546, 1.158]	0.011 [−0.207, 0.230]
	Total	2009*	0.74	0.048 [0.038, 0.058]	0.008 [−0.003, 0.018]

Note

The data span from 2000 to 2015 and the response variable considered is the sum of the counts per year per transect. The early‐onset group comprises all regions for which WNS was detected before 2012, the late‐onset group comprises regions for which WNS was detected after 2012, and the overall group is made of all regions. The table shows *N*/A in cases where the analysis found no significant breakpoint.

*** *p* < .001.

** *p* < .01.

* *p* < .05.

Piecewise regressions for big brown/silver‐haired bats showed marked changes in the separate regression estimates, like those of *Myotis*. In the early‐onset regions, the activity of the former species increased significantly (β = 0.22 ± 0.02, *p* = .01) from 2010 onwards (*SE* = 0.29, *p* < .001) instead of decreasing, one year following the beginning of declines in *Myotis* activity. But the breakpoints identified for the late‐onset and the province as a whole did not match any known threat that would have occurred around these years.

The breakpoint for red bats in both early‐onset region and in the province as a whole was in 2012 (*SE* = 0.27, *p* < .001) and their activity subsequently went back down (early: β = −0.69 ± 0.32, *p* = .01, overall: β = −0.67 ± 0.32, *p* = .01). When considering the impact of wind energy facilities, the breakpoint for red bats was in 2013 in both the northeastern regions close to wind energy facilities and those further away (Table 3); however, large intervals around the slope estimate after the breakpoint in the northeastern group make it inconclusive. Inspection of the residuals showed some linearity issues that were more marked after the breakpoint, mainly due to the low number of detections before 2004 and after 2013. In the case of hoary bats, their breakpoint was in 2012 in early‐onset regions (*SE* = 0.26, *p* < .001), as well as globally (*SE* = 0.29, *p* < .001) but examining the regressions from a windfarm perspective did not shed light on a relationship between breakpoint years and a known threat. The overall breakpoint for tricolored bats was in 2013 (*SE* = 0.41, *p* < .001), although there was very little activity in this species, either before or after 2013, and investigation of the residuals suggests linearity issues.

**Table 3:** Summary of the breakpoints and slopes found in piecewise regression for migratory species in the province of Quebec, Canada

Group	Species	Breakpoint		Slope before breakpoint [CI]	Slope after breakpoint [CI]
		Year	*SE*		
Northeastern	Hoary	2005*	0.84	−0.09 [−0.20, 0.02]	0.08 [0.07, 0.10]
	Red	2013***	3.33	0.34 [0.27, 0.42]	−32.51 [−2119, 2054]
Other transects	Hoary	2009***	0.36	0.09 [0.07, 0.12]	−0.09 [−0.11, −0.06]
	Red	2013***	0.22	0.30 [0.24, 0.36]	−0.73 [−1.07, −0.40]

Note

The data span from 2000 to 2015 and the response variable considered is the sum of the counts per year per transect. The northeastern group comprises regions closest to wind energy facilities, and it is compared to the other regions.

*** *p* < .001.

** *p* < .01.

* *p* < .05.

### 3.2 Association between NAO and local weather

**FIGURE 3:**
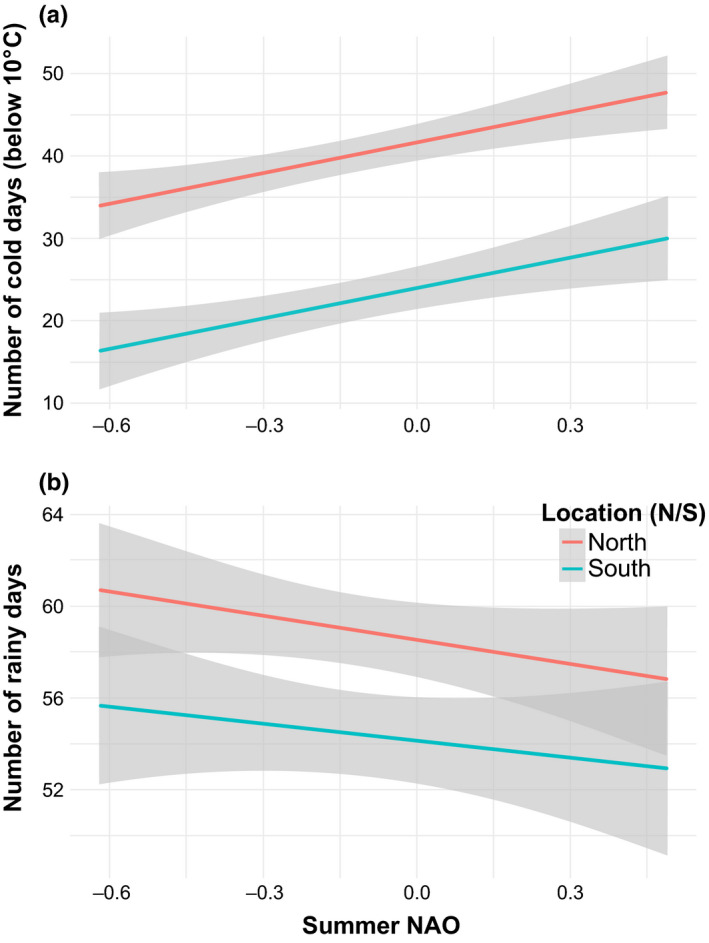
Based upon climate values that were recorded at weather stations closest to each of the 16 bat transects in Quebec (Canada), NAO‐positive summers were colder and drier than NAO‐negative summers. Indeed, as summer NAO increased, we observed a) an increase in the number of cold days below 10oC and b) a decrease in the number of rainy days that were recorded. Only the North–South difference was significant; hence, the lack of representation of East and West

**FIGURE 4:**
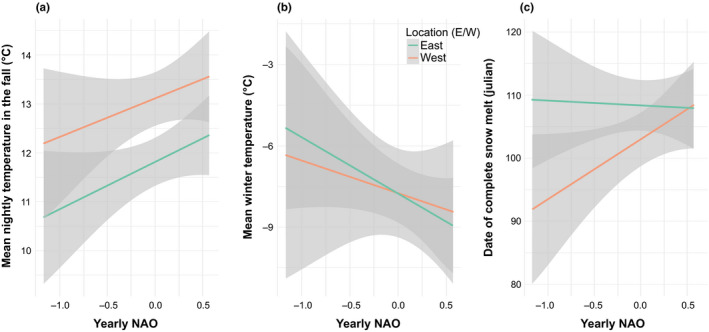
Based on climate values that were recorded at weather stations closest to each of the 16 bat transects in Quebec (Canada), NAO‐positive years translated into a) warmer nights in the autumn, b) colder winters, and c) later snowmelt in the West. The difference was only significant between East and West; hence, the lack of representation of North and South

Positive NAO values are generally associated with cold and dry conditions, as is generally the case in our study (Figure 3). Even if NAO‐positive years resulted in colder temperatures during winter, spring, and summer, autumn temperatures during our study were, in fact, warmer in positive than in NAO‐negative years (Figure 4). NAO‐positive summers tend to have more days during which the minimum daily temperature falls below 10°C, a threshold that is considered limiting to foraging (Anthony, Stack, & Kunz, 1981; Ciechanowski, Zając, Biłas, & Dunajski, 2007; Frick, Reynolds, & Kunz, 2010). The temperature in southern locations fell less frequently below 10°C, and they had fewer rainy days than northern locations. In the west, snow melts about 20 days earlier during NAO‐negative years than during NAO‐positive years.

### 3.3 Meta‐analyses of climatic variables

**FIGURE 5:**
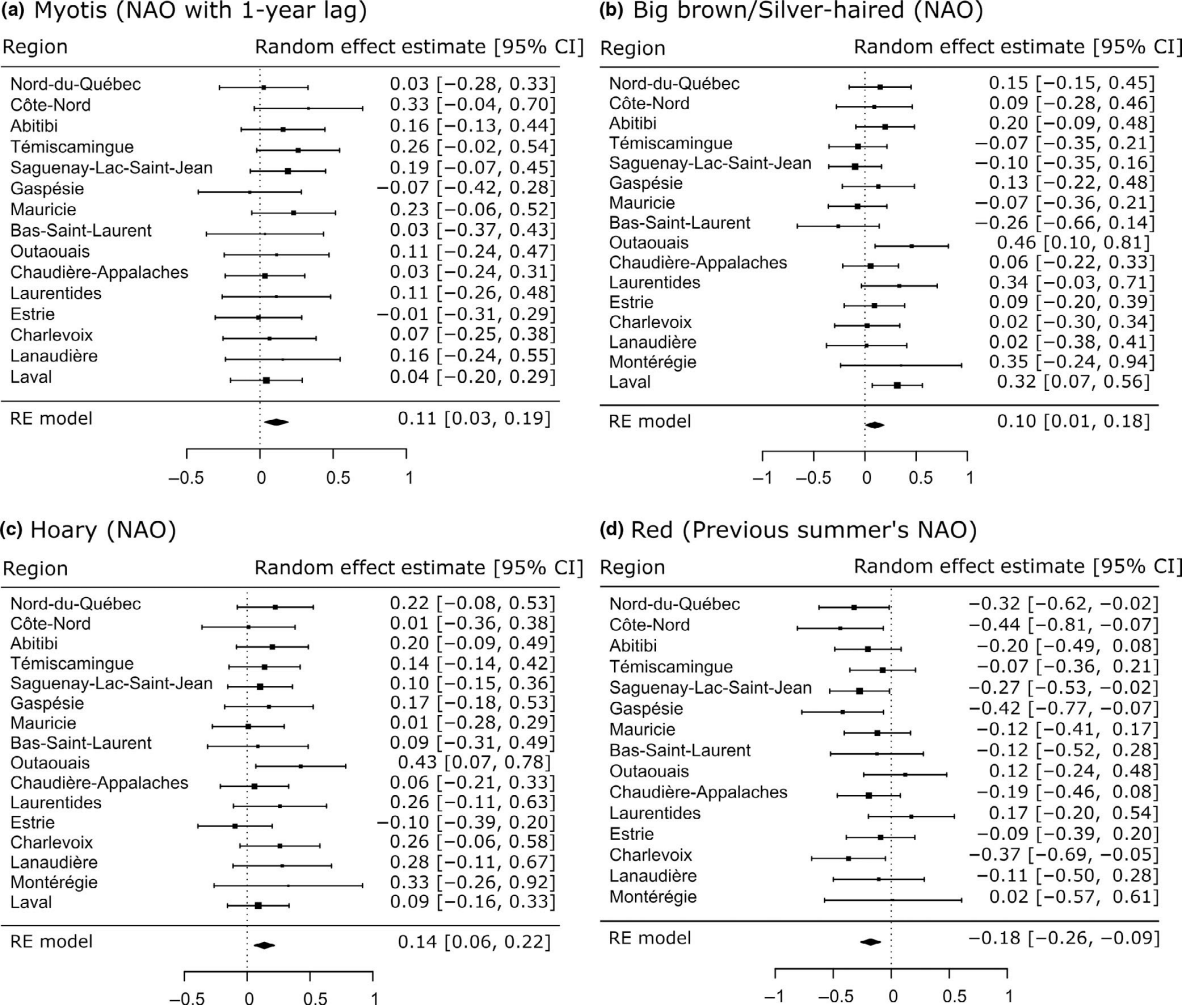
Meta‐analysis of climate and weather variables for each bat species that was detected during the 15‐year acoustic survey in Quebec, Canada. Effect sizes are computed for each for variable for which sufficient data were available in the 16 regions that were sampled

*Myotis* was positively correlated to the previous year's NAO index (*E* = 0.11, CI = 0.03–0.19, Figure 5), meaning high activity when the previous year was cold and dry, regardless of the current year's conditions. It was more specifically correlated to the NAO index during the winter that year (*E* = 0.09, CI = 0.01–0.16). The effect size of the yearly NAO index was small, but significant for big brown/silver‐haired (*E* = 0.10, CI = 0.01–0.18), and more specifically to the fall (*E* = 0.10, CI = 0.03–0.18) and winter (*E* = 0.10, CI = 0.01–0.19) and similar to hoary bats (yearly: *E* = 0.14, CI = 0.06–0.22, winter: *E* = 0.12, CI = 0.04–0.20), showing that those species were more active during NAO‐positive years, that is, having cold and dry conditions. Red bat activity was negatively correlated with the previous summer's NAO index (*E* = −0.18, CI = −0.26, −0.09), meaning low activity in the year following a cold and dry summer. They were also positively correlated to spring temperatures (*E* = 0.10, CI = 0.01, 0.20). The low count of tricolored bat calls made it difficult to detect any correlation between activity and climate conditions.

### 3.4 Effect of geographical location on the relationship between NAO index and activity

**FIGURE 6:**
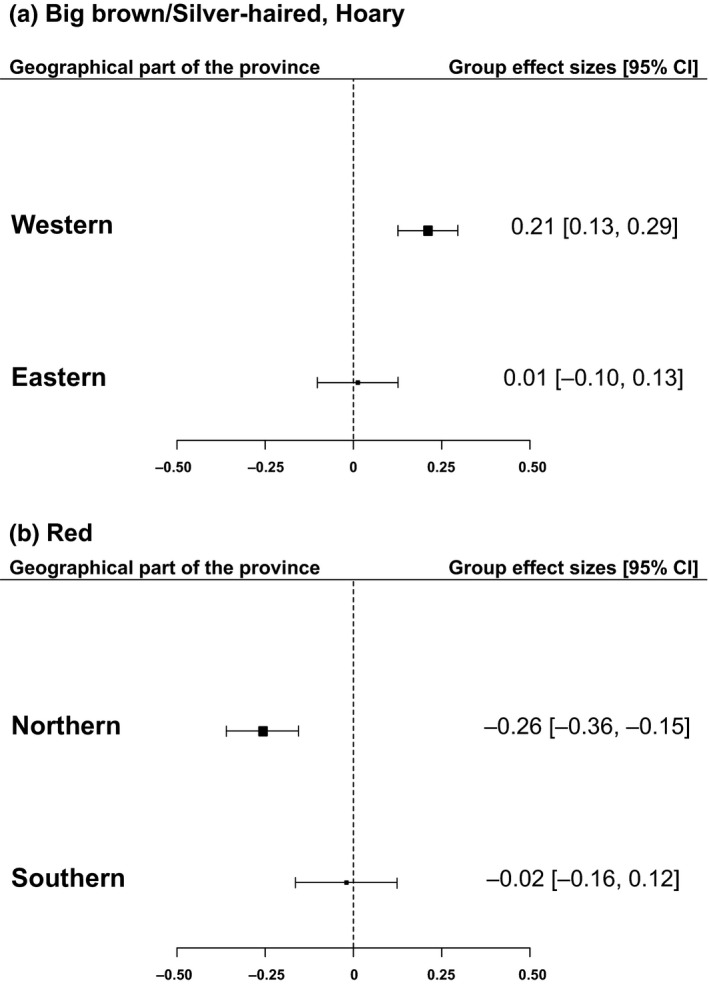
Group effect sizes of NAO index on big brown/silver‐haired and hoary bats located in eastern and western regions of Quebec, Canada. The groups are based upon geographical locations of the participating regions and the data cover the 15‐year span of the survey

The activity levels of big brown/silver‐haired and hoary bats were positively correlated to the current year's NAO index, and that effect was significantly different for the eastern and western regions of the province (*P*(*Q*_M_) < 0.0001). The effect of the NAO index on activity in western regions was significant and positive (*E* ^+^ = 0.21, CI = 0.13, 0.29), whereas it was not significantly different from zero in eastern regions (Figure 6). The effect of the NAO index on red bat activity also varied according to their geographical locations (*P*(*Q*_M_) < 0.001). The effect of the previous summer's NAO index was only significantly different from zero in northern regions (*E* ^+^ = −0.26, CI = −0.36, −0.15).

## 4 DISCUSSION

Our results support 3 of our 6 predictions and partly support 2 of them. We observed a decline in the activity of *Myotis* species after initial WNS detection in the regions. We only observed an increase in activity for big brown/silver‐haired bat activity after the initial detection of WNS, with a matching breakpoint in 2010 in early‐onset regions. Globally, we found that the constant increase in bat activity plateaued around 2009, which coincides with the breakpoints observed in *Myotis* and big brown/silver‐haired bats. We saw stochastic fluctuations modulated by climatic variables in all species except tricolored bats and warm falls appeared beneficial to most of them. However, contrary to our expectations, cold and dry winters are beneficial to hibernating species*.*

### 4.1 Effect of WNS and windfarms on bat activity

Our finding of a 79% drop in *Myotis* activity following the first assessment of WNS shows a decline just below those detected in populations the province, which suggested a 95% decline from hibernacula counts, and 94% from surveys in 10 maternity colonies (Équipe de Rétablissement des Chauves‐souris du Québec, 2019). They are consistent with reported capture rates (Francl, Ford, Sparks, & Brack, 2012; Ingersoll, Sewall, & Amelon, 2016; Moosman, Veilleux, Pelton, & Thomas, 2013) and other results from acoustic surveys, which reported a 75% *Myotis* decline following WNS introduction in one case (Frick, Pollock, et al., 2010) and a 72% decline in another (Brooks, 2011). However, cases where the decline occurred before the official detection suggest that WNS affected the region before it was detected by the local teams. Similarly to our assessments of big brown/silver‐haired bat activity, Morningstar et al. (2019) found a significant increase in big brown bat activity, coinciding with a decline of little brown bat activity following the onset of WNS in Ontario. Hauer, Powers, Mcnaughton, Paul, and Sewall (2019) also found a decline in *Myotis* captures and a 276% increase in big brown bat captures during WNS. However, Francl et al. (2012) found that capture rates of big brown bats in mist nets increased by only 17% following the first assessment of WNS, which was not significant compared to captures before WNS. Stable capture rates of big brown bats during WNS were also reported by Moosman et al. (2013) and Pettit and O’Keefe (2017). In our case, their activity more than doubled after the onset of WNS. Not only might they benefit from exploiting prey resources previously shared with *Myotis* as found by Morningstar et al. (2019), they may also have a higher availability of roosting (Thalken et al., 2018) and hibernating locations. Frank et al. (2014) found a 43% increase in the number of big brown bats hibernating in two WNS‐affected locations, which is in line with the high numbers of big brown bats found in the southern part of the province (Batwach.ca, unpublished data).

Migratory species like red and hoary bats are not affected by WNS (Bernard et al., 2015), which showed in our results. However, they are known to be negatively affected by wind energy production (Arnett et al., 2008). We found a significant breakpoint in 2012 when wind energy facilities started being built mostly in the northeastern part of the province. The differences in activity after 2012 between the northeastern and the rest of the transects were not significant for red bats, but the activity of hoary bats significantly decreased. However, when the breakpoint was 2013, the second part of the piecewise regression was made of only 3 years, which is a bare minimum to estimate a trend. Preliminary analysis of additional data from 2016 to 2018 indicates that a slow decrease might be a trend in hoary bats, while red bats had fluctuated back to 2012 activity in 2018 (unpublished data).

### 4.2 Association between NAO index and bat activity

Similarly to other studies (Brack, Stihler, Reynolds, Butchkoski, & Hobson, 2002; Thogmartin & McKann, 2014), we found climatic indices to be relevant predictors of bat activity, in the present case NAO index. Yet, the relationship between climatic conditions and bat activity did not conform to our prediction, as for both *Myotis* and big brown/silver‐haired bats we found an increase in bat activity during NAO‐positive years, that is, cold and dry conditions. The major difference being that the NAO effect was lagged for the Myotis but not for the big brown bat. Because we analyzed WNS separately from climate correlates, it is probable that the drastic changes in activity had an influence on the results of the meta‐analysis. However, meta‐analyses are generally robust to individual variations (Viechtbauer, 2015) and the fact that both *Myotis* and big brown/silver‐haired bats follow similar trends climate‐wise and opposite trends WNS‐wise indicates that the effect of separating the two analyses is minimal. In addition, the piecewise regression is designed to detect stable trends across years and would only be affected by NAO values increasing or decreasing in a linear fashion from or to the breakpoints, which would then be the same for all species.

In our study area, NOA‐positive years were associated with warm autumns, especially in the West, followed by cold winters and delayed snowmelt in the West. We, therefore, suggest that the possible positive effect of NOA‐positive years is likely attributed to advantageous autumn weather. Studies on Myotis suggested that fat deposition in September is particularly important, making up to 30% of prehibernating fat deposition (Ewing et al., 1970; Kunz et al., 1998). Moreover, juvenile bats tend to have less fat than adults in early autumn and to extend fat accumulation until mid‐October (Kunz et al., 1998). Kunz et al. (1998) also suggested that the capacity to store fat could be particularly limiting in northern latitudes, especially for young female reproduction and survival. In Québec province, September and October temperature can be quite variable, possibly allowing differential capacity to accumulate prehibernating fat.

Despite the warm autumns under NAO‐positive years, the positive relationship between the NAO index and bat activity is still surprising as it also involves colder winters and delayed springs. If bats hibernate in poorly tempered locations inside the hibernacula (Brack, 2007; Perry, 2013a), abrupt drops in temperature may trigger arousal (Davis & Reite, 1967) that can account for up to 75% of energy requirements during hibernation (Thomas & Geiser, 1997), causing depletion of fat reserves, and compromising survival and the success of ovulation and fertilization (Kunz et al., 1998). It is also possible that individuals who survived cold and dry winters benefited from those conditions lasting into spring, allowing them to delay their arousal and shorten the amount of time exposed to unstable spring conditions (Frick, Reynolds, et al., 2010; Norquay & Willis, 2014) a strategy used by males (Norquay & Willis, 2014).

Red bats are the only species for which activity was influenced by lagged values of the summer NAO index rather than by the yearly NAO index; their activity was low in northern regions when the previous summer was cold (NAO‐positive). As is the case in other migratory species (Cryan, 2003), lactating female red bats avoid torpor under low temperatures. Yet, red bats are known for a low degree of flexibility in their thermoregulatory response and high reproductive rate (Dunbar & Tomasi, 2006). During short periods of hibernation during the winter, they are known to benefit from the presence of leafy litter when the ambient temperature ranges below 10°C (Mormann & Robbins, 2007) to minimize energy expenditure, which allows them to hibernate in a wider range of temperatures (Perry, 2013b). If temperatures frequently drop below 10°C in the spring and early summer, they might suffer from higher energy expenditures if they cannot benefit from a protective leafy cover, resulting in poor reproduction rates.

## 5 CONCLUSIONS

In Quebec, the sharp decline in *Myotis* activity is consistent with the well‐documented impact of WNS. However, as Ingersoll et al. (2016) have noted, several factors might be at play in stochastic changes and regional population declines. The current state of conservation and biodiversity research has been increasingly centered around climate change, and as we have found, there is indeed a relationship between bats and climate. This being said, we want to echo a call from Titeux, Henle, Mihoub, and Brotons (2016) for a more balanced research agenda that puts in perspective and integrates a wider range of threats. Eventual northward shifts in wintering ranges related to climate change have been predicted in little brown bats (Humphries, Thomas, & Speakman, 2002) and Indiana bats (Thogmartin, King, Szymanski, & Pruitt, 2012), but these shifts may never occur if the species become locally extirpated.

We show an example where competition release following the collapsing of bats species vulnerable to an introduced fungus may create rapid shifts in community compositions if combined with the effect of human‐made threats like windfarms to migrating bat species. Thus, they should be considered together with a wider range of potential threats in long‐term monitoring, in conjunction with the impact of climate change on conservation efforts.

## AUTHORS’ CONTRIBUTION

**Julie Faure‐Lacroix:** Conceptualization (equal); Data curation (lead); Formal analysis (lead); Investigation (lead); Methodology (equal); Project administration (equal); Writing‐original draft (lead); Writing‐review & editing (lead). **André Desrochers:** Conceptualization (equal); Data curation (supporting); Formal analysis (supporting); Funding acquisition (lead); Investigation (supporting); Methodology (supporting); Project administration (equal); Supervision (lead); Writing‐original draft (equal); Writing‐review & editing (equal). **Louis Imbeau:** Conceptualization (equal); Data curation (supporting); Formal analysis (supporting); Funding acquisition (equal); Investigation (supporting); Methodology (supporting); Project administration (equal); Supervision (equal); Writing‐original draft (equal); Writing‐review & editing (equal). **Anouk Simard:** Conceptualization (equal); Data curation (equal); Formal analysis (supporting); Funding acquisition (lead); Investigation (equal); Methodology (equal); Project administration (equal); Supervision (equal); Writing‐original draft (equal); Writing‐review & editing (equal).

## Data Availability

Upon acceptance of this manuscript, all data will be uploaded to FigShare and made available to the public. https://figshare.com/authors/Julie_Faure‐Lacroix/6542219
